# Association of lipid, inflammatory, and metabolic biomarkers with age at onset for incident cardiovascular disease

**DOI:** 10.1186/s12916-022-02592-x

**Published:** 2022-11-10

**Authors:** Xue Tian, Shuohua Chen, Yingting Zuo, Yijun Zhang, Xiaoli Zhang, Qin Xu, Yanxia Luo, Shouling Wu, Anxin Wang

**Affiliations:** 1 Department of Neurology, Beijing Tiantan Hospital, Capital Medical University, No.119 South 4th Ring West Road, Fengtai District, Beijing, 100070 China; 2 China National Clinical Research Center for Neurological Diseases, Beijing Tiantan Hospital, Capital Medical University, Beijing, China; 3 Department of Epidemiology and Health Statistics, School of Public Health, Capital Medical University, No.10 Xitoutiao, You’anmen Wai, Fengtai District, Beijing, 100069 China; 4 Beijing Municipal Key Laboratory of Clinical Epidemiology, Beijing, China; 5 Department of Cardiology, Kailuan Hospital, North China University of Science and Technology, 57 Xinhua East Rd, Tangshan, 063000 China

**Keywords:** Cardiovascular disease, Risk factors, Young adults, Age-related risk

## Abstract

**Background:**

Risk profiles for premature cardiovascular disease (CVD) are unclear. This study aimed to examine baseline risk profiles for incident CVD by age at onset in Chinese population.

**Methods:**

A total of 97,841 participants without CVD were enrolled from the Kailuan cohort study. Four age groups were examined (< 55, 55 to < 65, 65 to < 75, and ≥ 75 years) for CVD onset. Risk profiles included clinical, lipid, metabolic, and inflammatory risk factors and biomarkers.

**Results:**

Of the clinical factors, diabetes was associated with the highest relative risk for incident CVD in participants younger than 55 years (sub-distributional hazard ratio [sHR], 4.08; 95% confidence interval [CI], 3.47–4.80). Risk factors that were also noted for CVD onset in participants younger than 55 years included hypertension, metabolism syndrome, overweight or obese, dyslipidemia, and smoking. Among the biomarkers, insulin resistance measured by triglyceride-glucose index had the highest sHR (1.42; 95% CI, 1.35–1.49) for CVD in participants younger than 55 years. In comparison, weaker but significant associations with CVD in participants younger than 55 years were noted for most lipids, metabolic biomarkers, and inflammatory biomarkers. Most risk factors and biomarkers had associations that attenuated with increasing age at onset. Some biomarkers had similar CVD age association, while a few had no association with CVD onset at any age.

**Conclusions:**

These findings showed that diabetes and insulin resistance, in addition to hypertension, metabolism syndrome, overweight or obese, dyslipidemia, and smoking, appeared to be the strongest risk factors for premature onset of CVD, and most risk factors had attenuated relative rates at older ages.

**Supplementary Information:**

The online version contains supplementary material available at 10.1186/s12916-022-02592-x.

## Background

Cardiovascular disease (CVD) is the leading cause of premature death worldwide [1]. Premature CVD generally refers to having a history of CVD events before the age of 55 and 65 for men and women, respectively [2, 3]. Despite advances in CVD prevention and management, outcomes among young adults have been suboptimal. The clinical significance of identifying individuals at risk of premature CVD in reducing the burden of premature morbidity and mortality is now increasingly being recognized, due to the incidence of CVD among young adults has been stagnating or increasing [4–7]. However, evidence on characterization of biomarker profiles that identify premature CVD has been inadequate.

The reasons for suboptimal outcomes are multifactorial, including temporal trends in age-based differences in risk factors, clinical presentation, acute management, or use of preventive therapies [8–11]. Literatures regarding the determinants on premature CVD were limited. Most biomarker studies of premature CVD have been cross-sectionally designed in the European population and reported differences in the levels of serum lipids for premature vs conventional CVD, as well as differences by sex for premature CVD [11–14]. Emerging evidence suggested that CVD risk may also be associated with novel biomarkers related to lipoprotein subfractions, inflammation, and metabolic pathways, and not merely with levels of standard risk factors [15–17]. However, characterization of biomarker profiles that identify premature CVD has been inadequate. Recently, the Women’s Health Study investigated more than 50 risk factors and biomarkers related to coronary heart disease among women [18]. To our knowledge, there was no large prospective study on the association of an extensive panel of novel and traditional biomarkers according to age at the time of incident CVD among Chinese population.

To address these knowledge gaps, we investigated the relevant risk of clinical risk factors and lipid, metabolic, and inflammation biomarkers with incident CVD in a large community-based prospective cohort.

## Methods

### Study population

The study population were from the Kailuan study, which is an ongoing prospective cohort study conducted in Tangshan, China. The details on Kailuan study have been previously described [19–21]. Briefly, since June 2006, a total of 101,510 participants (81,110 men and 20,400 women, aged 18–98 years) were enrolled in the first survey from 11 hospitals and underwent questionnaire assessments, clinical examinations, and laboratory tests. All participants were followed biennially to update their status on the aforementioned parameters. In the present study, we excluded participants with a history of myocardial infraction or stroke (*n* = 3669) at baseline; ultimately, a total of 97,841 participants were included in the current analysis. The study was performed according to the guidelines of the Helsinki Declaration and was approved by the Ethics Committee of Kailuan General Hospital (approval number: 2006–05) and Beijing Tiantan Hospital (approval number: 2010–014-01). All the participants agreed to take part in the study and provided written informed consent.

### Incident CVD ascertainment

Incident CVD was a composite of first stroke or myocardial infarction. Assessment of CVD has been described previously [21–23]. The database of CVD diagnoses was obtained from the Municipal Social Insurance Institution and Hospital Discharge Register and was updated annually. An expert panel collected and reviewed the annual discharge records from 11 hospitals in the Kailuan community to identify patients who were suspected of CVD. Incident stroke was diagnosed based on neurological signs, clinical symptoms, and neuroimaging tests, including computed tomography or magnetic resonance, according to the World Health Organization criteria [24]. Myocardial infarction was diagnosed according to the criteria of the World Health Organization based on clinical symptoms, changes in the serum concentrations of cardiac enzymes and biomarkers, and electrocardiographic results [22, 25].

### Risk factors assessment

Baseline risk factors were collected via a standardized questionnaire by trained staff, including age, sex, education level, family income, physical activity, smoking, alcohol intake, and medical history (hypertension, diabetes, and dyslipidemia). Educational level was classified as illiterate or primary school, middle school, and high school or above. Income was categorized into > 800 and ≤ 800 yuan/month. Smoking and alcohol intake habits were stratified into never, former, or current. Physical activity included both physical activity in leisure time and at work and then was categorized as active physical activity (≥ 80 min per week at leisure or manual work) physical inactivity (< 80 min per week, or none at leisure time, or mental work) [26]. Body mass index (BMI) was calculated as weight in kilograms divided by the height in meters squared and were categorized as overweight (BMI 25.0 to < 28.0) and obsess (BMI ≥ 28.0) [27]. Blood pressure was measured in the seated position using a mercury sphygmomanometer, and the mean results of three measurements of systolic blood pressure (SBP) and diastolic blood pressure (DBP) were recorded. Hypertension was defined as SBP ≥ 140 mm Hg or DBP ≥ 90 mm Hg, any use of the antihypertensive drug, or a self-reported history of hypertension [28]. Diabetes was defined as fasting blood glucose (FBG) ≥ 7.0 mmol/L, any use of glucose-lowering drugs, or a self-reported history of diabetes. Dyslipidemia was defined as any self-reported history or use of lipid-lowering drugs, or total cholesterol (TC) ≥ 5.17 mmol/L or triglyceride (TG) ≥ 1.69 mmol/L or low-density lipoprotein cholesterol (LDL-C) ≥ 3.62 mmol/L or high-density lipoprotein cholesterol (HDL-C) ≤ 1.04 mmol/L. Metabolic syndrome was defined according to the ATP-III criteria [29].

### Biomarker measurements

Fasting blood samples were collected in the morning after an 8- to 12-h overnight fast and transfused into vacuum tubes containing EDTA. Plasma was separated from blood immediately and stored at 4 °C. All the blood samples were analyzed using an auto-analyzer (Hitachi 747, Hitachi, Tokyo, Japan) on the day of the blood draw. The biochemical indicators included FBG, serum lipids (TC, TG, LDL-C, HDL-C, TC/HDL-C, TG/HDL-C, non-HDL-C, remnant cholesterol [calculated as TC-LDL-C-HDL-C]), serum creatinine, high-sensitivity C-reactive protein (hs-CRP), white blood cell count, neutrophil count, and platelet count. The triglyceride-glucose index (TyG) was calculated as ln (fasting TG [mg/dl] × FBG [mg/dl]/2) [30]. Estimated glomerular filtration rate (eGFR) was calculated by the Chronic Kidney Disease Epidemiology Collaboration creatinine equation [31].

### Statistical analysis

We divided the age at CVD onset into 4 age groups (< 55, 55 to < 65, 65 to < 75, and ≥ 75 years) and participants contributed to advancing age groups over time until the occurrence of incident CVD or censoring (death or the end of the follow-up), and calculated CVD incidence rates. The baseline characteristics were presented as mean ± standard deviation (SD) or frequency with percentage as appropriate. Differences in the characteristics across 4 age categories were tested using analysis of variance or the Kruskal–Wallis test for continuous variables according to distribution, and using chi-square for categorical variables.

Considering the proportion of non-CVD death was over 5%, we used stratified competing risk models with the counting process method, stratified by the 4 age groups, in which non-CVD death was regarded as a competing risk event. We estimated adjusted sub-distributional hazard ratio (sHR) with 95% confidence interval (CI) for per SD increment of each biomarker and for clinical categories of risk factors with clinical cutoff points. The basic adjusted model included sex, educational level, and family income, then each risk factor was added individually to the basic adjusted model stratified by age groups. In additional analyses, we examine the associations between risk factors and incident CVD in the models that included covariates mentioned above plus the following additional covariates (physical activity, smoking, drinking, BMI, hypertension, diabetes, dyslipidemia, SBP, and DBP). The proportional hazard assumptions were evaluated by visualization of Schoenfeld residuals, and no potential violation was observed. The population-attributable risk for clinical risk factors was calculated with a method previously described [32]. Likelihood ratio tests were used to evaluate the interaction between each individual risk factor and age groups in the basic adjusted model.

All analyses were performed using SAS version 9.4 (SAS Institute, Cary, NC). All statistical tests were 2-sided, and *P* < 0.05 was considered statistically significant.

## Results

### Baseline characteristics

Of 97,841 participants included in the analysis, 90,197 (92.24%) participants did not develop CVD and incident CVD occurred in 7587 patients (7.76%) during the study period. Most baseline characteristics differed between CVD cases and non-cases (Table 1, Additional file 1: Table S1) and across age groups (Additional file 1: Table S2). The prevalence of most clinical risk factors and levels of serum lipids and metabolic and inflammatory biomarkers were higher in cases than in non-cases.

**Table 1:** Baseline characteristics of the participants with and without CVD

Characteristics	No. (%)				Noncases (*n* = 90,197)
	Incidence CVD				
	At age < 55 y (*n* = 1241)	At 55 to < 65 y (*n* = 2693)	At 65 to < 75 y (*n* = 2273)	At age ≥ 75 y (*n* = 1380)	
Clinical risk factors					
Age, y	44.29 ± 5.56	53.37 ± 4.20	62.50 ± 4.95	72.66 ± 5.51	50.96 ± 12.59
Men, *n* (%)	1097 (88.40)	2399 (89.08)	2028 (89.22)	1246 (90.29)	71,048 (78.77)
High school or above, *n* (%)	55 (4.49)	66 (2.54)	56 (2.62)	36 (3.06)	6389 (7.371)
Income > 800 yuan/month, *n* (%)	143 (11.69)	296 (11.44)	254 (11.92)	191 (16.26)	12,427 (14.34)
Current smoker, *n* (%)	570 (46.68)	1112 (42.92)	767 (35.62)	302 (24.34)	29,577 (33.85)
Current drinker, *n* (%)	546 (44.64)	1000 (38.60)	730 (33.97)	354 (28.55)	32,760 (37.48)
Physical inactivity, *n* (%)	133 (10.88)	229 (8.88)	111 (5.23)	53 (4.51)	7631 (8.82)
Hypertension, *n* (%)	720 (58.02)	1734 (64.39)	1556 (68.46)	946 (68.55)	36,969 (40.99)
Diabetes, *n* (%)	175 (14.10)	486 (18.05)	434 (19.09)	209 (15.14)	7438 (8.25)
Dyslipidemia, *n* (%)	557 (44.88)	1183 (43.93)	980 (43.11)	513 (37.17)	31,145 (34.53)
Metabolism syndrome, *n* (%)	249 (20.06)	642 (23.84)	583 (25.65)	316 (22.90)	11,967 (13.27)
Body mass index, kg/m.^2^	26.18 ± 3.61	25.91 ± 3.46	25.54 ± 3.37	25.12 ± 3.42	24.96 ± 3.49
Overweight or obese, *n* (%)	748 (60.27)	1562 (58.00)	1224 (53.85)	675 (48.91)	42,098 (46.67)
Systolic blood pressure, mm Hg	138.26 ± 23.68	141.36 ± 22.75	144.56 ± 22.96	144.45 ± 21.27	129.64 ± 20.41
Diastolic blood pressure, mm Hg	90.05 ± 14.57	89.71 ± 13.18	88.00 ± 11.95	84.04 ± 10.73	82.96 ± 11.58
Lipids profile					
Total cholesterol, mmol/L	5.21 ± 1.15	5.12 ± 1.18	5.11 ± 1.22	5.02 ± 1.20	4.93 ± 1.14
Triglycerides, mmol/L	2.05 ± 1.62	1.97 ± 1.58	1.81 ± 1.45	1.63 ± 1.21	1.66 ± 1.36
LDL cholesterol, mmol/L	2.44 ± 0.93	2.40 ± 0.94	2.39 ± 1.14	2.33 ± 1.08	2.34 ± 0.90
HDL cholesterol, mmol/L	1.54 ± 0.40	1.55 ± 0.41	1.57 ± 0.44	1.59 ± 0.49	1.55 ± 0.40
Total/HDL cholesterol	3.58 ± 1.37	3.48 ± 1.12	3.48 ± 1.55	3.39 ± 1.47	3.39 ± 3.26
Triglyceride/HDL cholesterol	1.44 ± 1.37	1.36 ± 1.18	1.28 ± 1.48	1.13 ± 1.17	1.17 ± 2.26
Non-HDL-C, mmol/L	3.67 ± 1.10	3.56 ± 1.17	3.53 ± 1.21	3.42 ± 1.21	3.38 ± 1.11
Remnant cholesterol, mmol/L	1.23 ± 1.15	1.16 ± 1.25	1.14 ± 1.32	1.10 ± 1.36	1.04 ± 1.15
Metabolic and Inflammatory					
Fasting blood glucose, mmol/L	5.86 ± 2.17	6.04 ± 2.29	5.92 ± 2.18	5.69 ± 1.99	5.43 ± 1.61
Triglyceride-glucose index	8.90 ± 0.75	8.90 ± 0.73	8.81 ± 0.71	8.69 ± 0.68	8.64 ± 0.69
eGFR, ml/min/1.73/m.^2^	86.25 ± 21.83	80.9 ± 23.98	76.6 ± 27.72	71.43 ± 40.79	82.56 ± 25.47
Creatinine, μmol/L	94.50 ± 41.98	94.74 ± 40.29	93.55 ± 33.05	95.49 ± 35.58	91.56 ± 29.85
Serum uric acid, μmol/L	301.01 ± 88.77	295.31 ± 84.63	306.58 ± 89.47	314.79 ± 94.12	287.85 ± 83.18
Hs-CRP, mg/L	2.49 ± 5.32	2.66 ± 4.60	3.29 ± 7.05	3.94 ± 8.60	2.33 ± 6.48
White blood cell count, *10^9^/L	7.20 ± 1.98	7.04 ± 2.77	7.10 ± 13.09	6.50 ± 2.13	6.83 ± 9.86
Neutrophil count, *10^9^/L	4.34 ± 1.50	4.18 ± 1.71	4.13 ± 2.36	3.91 ± 1.29	3.97 ± 2.91
Platelet, *10^9^/L	219.23 ± 82.37	205.74 ± 56.82	198.56 ± 79.37	189.93 ± 78.41	211.22 ± 746.16
Red blood cell count, *10^9^/L	4.96 ± 0.50	4.89 ± 0.48	5.37 ± 16.04	4.67 ± 0.48	5.05 ± 26.46

Noncases did not develop cardiovascular disease during follow-up

*CVD* Cardiovascular disease, *eGFR* Estimated glomerular filtration rate, *HDL* High-density lipoprotein, *hs-CRP* High-sensitivity C-reactive protein, *LDL* Low-density lipoprotein

During median follow-up of 12.99 years, CVD incidence per 1000 person-years ranged from 4.69 (95% CI, 4.44–4.96) for CVD onset less than 55 years to 7.50 (95% CI, 7.11–7.90) for CVD onset at 75 years or older (Additional file 1: Table S3).

### Clinical risk factors

**Fig. 1:**
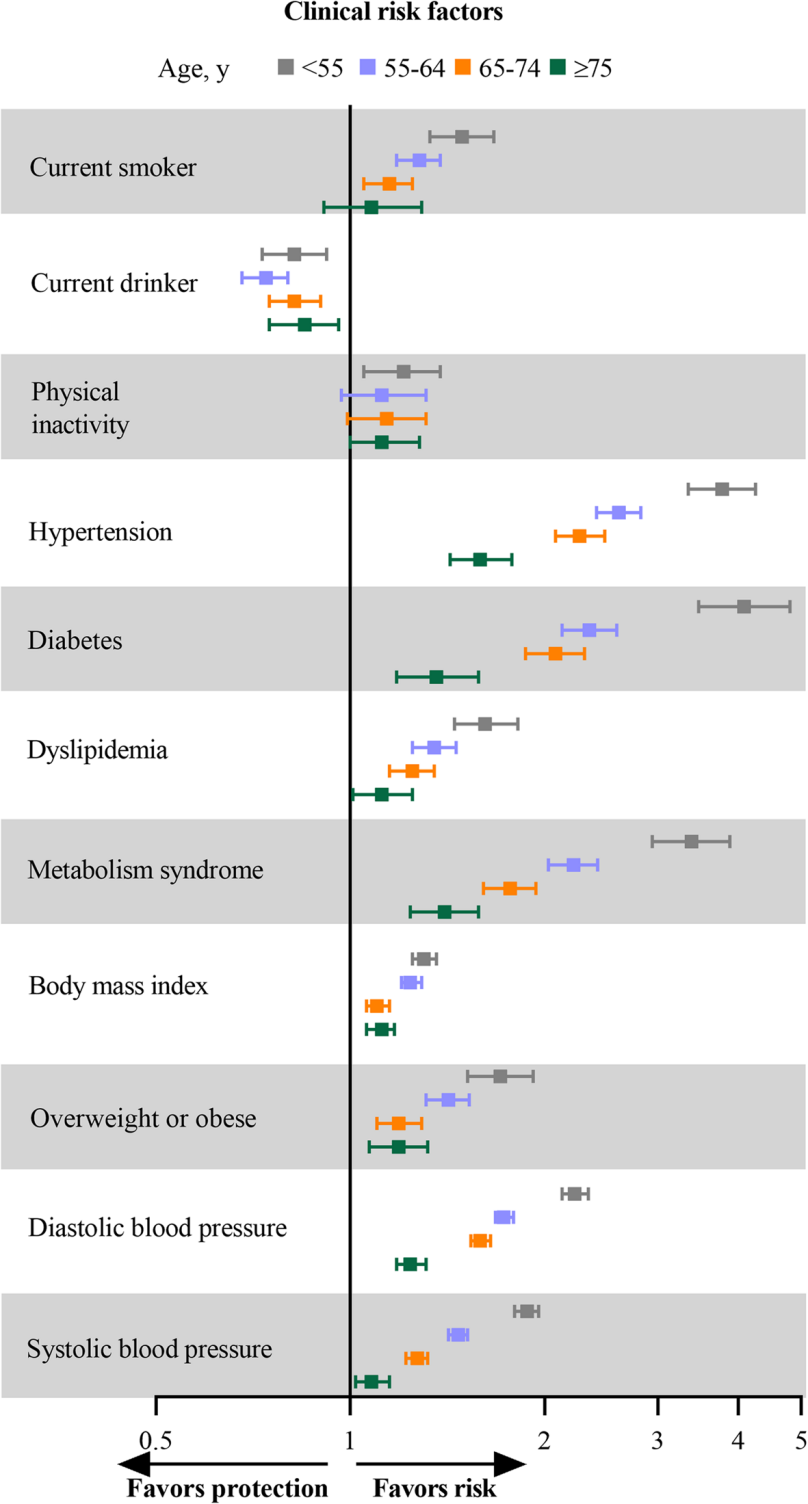
Sub-distributional hazard ratios with 95% confidence intervals for the association between clinical risk factors and risk of cardiovascular disease in different CVD-onset age groups. Model was adjusted for gender, educational level, and family income, and interactions between the risk factors of interest and age groups

Diabetes had the highest relative risk for incident CVD onset less than 55 years; the adjusted sHR was 4.08 (95% CI, 3.47–4.80). For incident CVD onset 55 years or older, hypertension had the highest relative risk; the adjusted sHR was 2.61 (95% CI, 2.41–2.82) and attenuated with age, with a risk of 1.59 (95% CI, 1.43–1.78) at onset in those 75 years or older. Increased risks were also noted for CVD onset in participants younger than 55 years for metabolism syndrome (sHR, 3.38; 95% CI, 2.94–3.88), overweight or obese (sHR, 1.71; 95% CI, 1.52–1.92), dyslipidemia (sHR, 1.62; 95% CI, 1.45–1.82), and smoking (sHR, 1.49; 95% CI, 1.33–1.67), which also attenuated with age (Fig. 1 and Table 2).

**Table 2:** Associations of risk factors with incident CVD by age at onset

	Incident CVD, adjusted sHR (95%CI)				*P* for interaction
	At age < 55 y	At age 55 to 65 y	At age 65 to 75 y	At age ≥ 75 y	
Clinical risk factors					
Current smoker	1.49(1.33–1.67)	1.28(1.18–1.38)	1.15(1.05–1.25)	1.08(0.91–1.29)	0.0006
Current drinker	0.82(0.73–0.92)	0.74(0.68–0.80)	0.82(0.75–0.90)	0.85(0.75–0.96)	0.8373
Physical inactivity	1.21(1.05–1.38)	1.12(0.97–1.31)	1.14(0.99–1.31)	1.12(1.00–1.28)	0.0844
Hypertension	3.77(3.34–4.25)	2.61(2.41–2.82)	2.27(2.08–2.48)	1.59(1.43–1.78)	< 0.0001
Diabetes	4.08(3.47–4.80)	2.35(2.13–2.59)	2.08(1.87–2.31)	1.36(1.18–1.58)	< 0.0001
Dyslipidemia	1.62(1.45–1.82)	1.35(1.25–1.46)	1.25(1.15–1.35)	1.12(1.01–1.25)	< 0.0001
Metabolism syndrome	3.38(2.94–3.88)	2.22(2.03–2.42)	1.77(1.61–1.94)	1.40(1.24–1.58)	< 0.0001
BMI, per SD increment	1.30(1.25–1.36)	1.24(1.20–1.29)	1.10(1.06–1.15)	1.12(1.06–1.17)	< 0.0001
Overweight or obese	1.71(1.52–1.92)	1.42(1.31–1.53)	1.19(1.10–1.29)	1.19(1.07–1.32)	< 0.0001
Systolic BP, per SD increment	2.22(2.12–2.34)	1.73(1.67–1.78)	1.58(1.52–1.63)	1.24(1.18–1.30)	< 0.0001
Diastolic BP, per SD increment	1.88(1.80–1.96)	1.47(1.42–1.51)	1.27(1.22–1.32)	1.09(1.03–1.15)	< 0.0001
Lipids, per SD increment					
Total cholesterol	1.33(1.27–1.38)	1.10(1.06–1.14)	1.07(1.03–1.12)	1.07(1.02–1.13)	< 0.0001
Triglycerides	1.15(1.11–1.19)	1.10(1.07–1.13)	1.08(1.05–1.12)	1.09(1.03–1.15)	0.0193
LDL cholesterol	1.05(0.98–1.14)	1.01(0.97–1.06)	1.04(1.00–1.08)	1.03(0.99–1.08)	0.8382
HDL cholesterol	1.14(1.07–1.21)	1.04(1.00–1.09)	1.04(1.00–1.08)	0.98(0.93–1.03)	0.0023
Total/HDL cholesterol	1.03(1.01–1.05)	1.04(1.00–1.07)	1.00(0.99–1.01)	1.01(1.00–1.02)	0.0549
Triglycerides/HDL cholesterol	1.01(1.00–1.01)	1.14(1.09–1.19)	1.07(1.03–1.11)	1.04(1.01–1.07)	< 0.0001
Non–HDL cholesterol	1.32(1.26–1.38)	1.09(1.05–1.13)	1.06(1.01–1.10)	1.08(1.03–1.14)	< 0.0001
Remnant cholesterol	1.31(1.25–1.38)	1.08(1.04–1.12)	1.02(0.98–1.06)	1.05(0.99–1.10)	< 0.0001
Metabolic, per SD increment					
Fasting blood glucose	1.31(1.24–1.38)	1.22(1.19–1.25)	1.16(1.13–1.19)	1.09(1.05–1.14)	< 0.0001
Triglyceride-glucose index	1.42(1.35–1.49)	1.26(1.22–1.31)	1.19(1.15–1.24)	1.14(1.09–1.21)	< 0.0001
eGFR	0.78(0.73–0.83)	0.79(0.75–0.84)	0.74(0.67–0.81)	0.96(0.85–1.09)	0.0030
Creatinine	1.00(0.93–1.06)	1.04(1.01–1.07)	1.07(1.04–1.11)	1.04(1.00–1.08)	0.1459
Serum uric acid	1.14(1.08–1.21)	1.10(1.05–1.14)	1.18(1.13–1.23)	1.07(1.02–1.12)	0.0076
Inflammatory, per SD increment					
Hs–CRP	1.12(1.08–1.17)	1.03(1.01–1.05)	1.06(1.03–1.09)	1.03(1.00–1.05)	0.0001
White blood cell count	1.07(1.01–1.12)	1.00(0.99–1.01)	1.02(0.99–1.04)	0.97(0.94–1.00)	0.1470
Neutrophil count	1.07(1.05–1.10)	1.03(1.01–1.04)	1.06(1.04–1.09)	1.00(0.97–1.03)	0.0248
Platelet	1.00(0.99–1.00)	0.44(0.28–0.69)	0.68(0.37–1.24)	0.96(0.55–1.65)	0.0022
Red blood cell count	0.00(0.00–0.86)	1.01(0.98–1.04)	1.01(0.98–1.04)	0.97(0.94–1.00)	0.0708

sHRs (95% CI) were obtained from stratified competing risk models with non-CVD death as a competing risk, adjusted for gender, educational level, and family income, and interactions between the risk factors of interest and age groups

*CI* Confidence interval, *CVD* Cardiovascular disease, *eGFR* Estimated glomerular filtration rate, *HDL* High-density lipoprotein, hs-*CRP* High-sensitivity C-reactive protein, *LDL* Low-density lipoprotein, *SD* Standard deviation, *sHR* Sub-distributional hazard ratio

### Lipids profiles

**Fig. 2:**
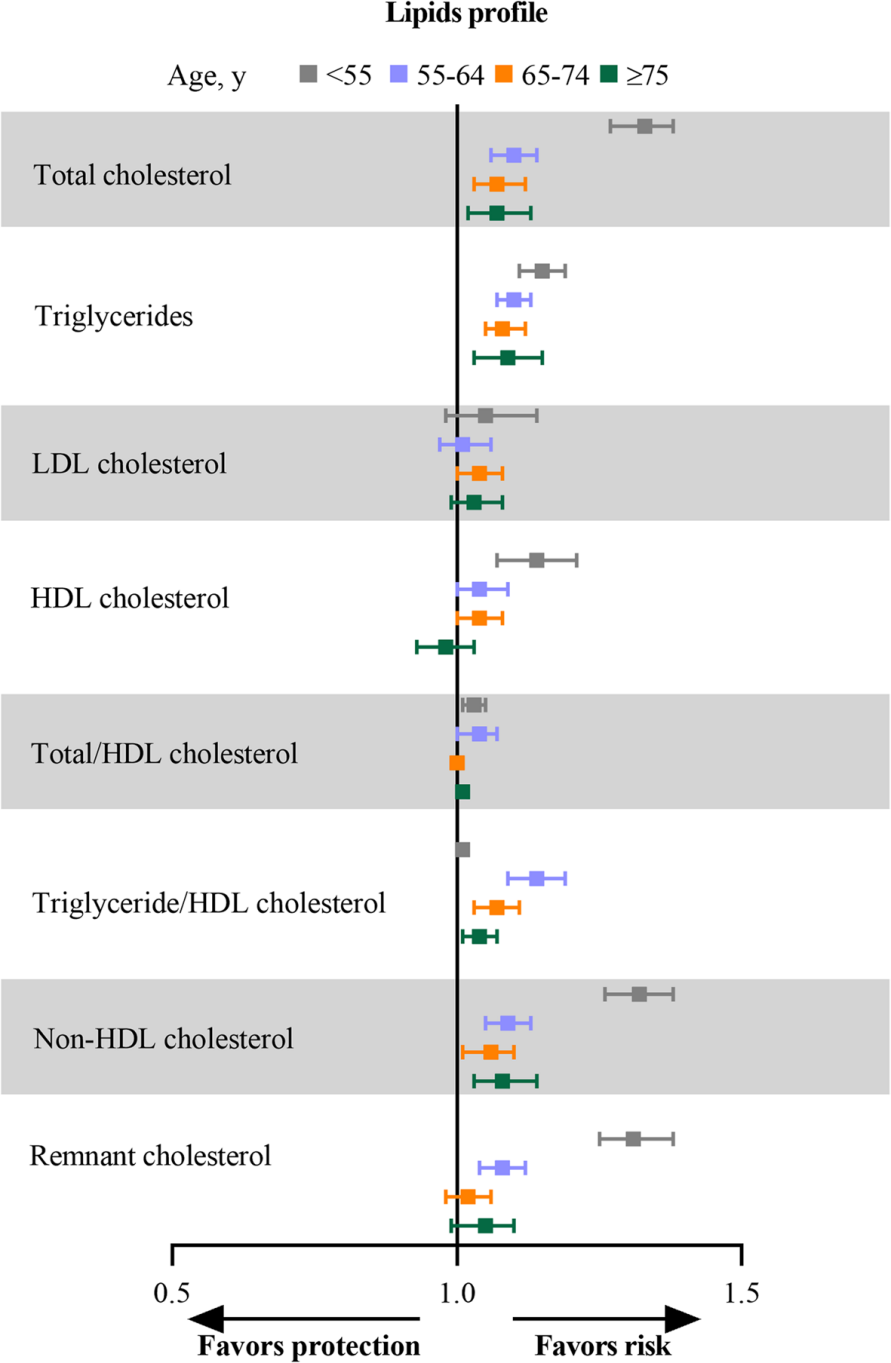
Sub-distributional hazard ratios with 95% confidence intervals for the association between lipids and risk of cardiovascular disease in different CVD-onset age groups. Model was adjusted for gender, educational level, and family income, and interactions between the risk factors of interest and age groups. Abbreviations: LDL, low-density lipoprotein; HDL, high-density lipoprotein

Significant associations with CVD onset in participants younger than 55 years were noted (per SD increase) for TC (sHR, 1.33; 95% CI, 1.27–1.38), TG (sHR, 1.15; 95% CI, 1.11–1.19), HDL-C (sHR, 1.14; 95% CI, 1.07–1.21), non-HDL-C (sHR, 1.32; 95% CI, 1.26–1.38), and remnant cholesterol (sHR, 1.31; 95% CI, 1.25–1.38) and attenuated with age. These biomarkers showed associations with incident CVD in almost all age groups. However, no significant difference was noted for LDL-C and TC/HDL-C with CVD by increasing age (Fig. 2 and Table 2).

### Metabolic and inflammatory biomarkers

**Fig. 3:**
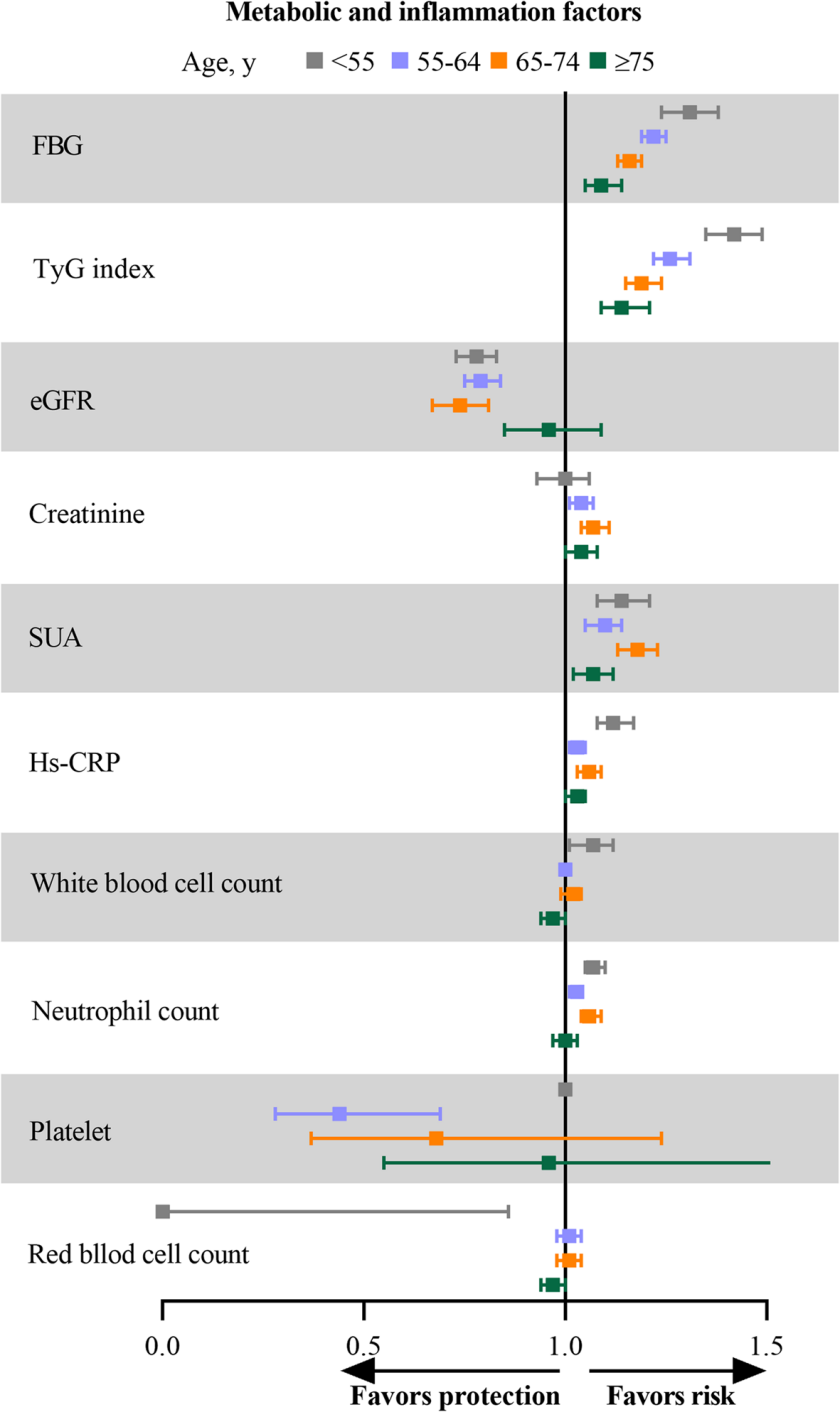
Sub-distributional hazard ratios with 95% confidence intervals for the association of metabolic and inflammatory biomarkers with risk of cardiovascular disease in different CVD-onset age groups. Model was adjusted for gender, educational level, and family income, and interactions between the risk factors of interest and age groups. Abbreviations: eGFR, estimated glomerular filtration rate; FBG, fasting blood glucose; hs-CRP, high sensitivity C-reactive protein; SUA, serum uric acid; TyG index, triglyceride glucose index

The TyG index had the highest relative risk of incident CVD onset at any age, for CVD onset younger than 55 years; the adjusted sHR for per SD increase was 1.42 (95% CI, 1.35–1.49) and attenuated with age, with a risk of 1.14 (95% CI, 1.09–1.21) at onset in those 75 years or older. Other biomarkers, including FBG (sHR, 1.31; 95% CI, 1.24–1.38), SUA (sHR, 1.154; 95% CI, 1.08–1.21), hs-CRP (sHR, 1.12; 95% CI, 1.08–1.17), and neutrophil count (sHR, 1.07; 95% CI, 1.04–1.12), showed positive associations with incident CVD; eGFR (sHR, 0.78; 95% CI, 0.73–0.83) showed negative association with incident CVD which all attenuated with age (Fig. 3 and Table 2).

### Additional analyses

We examined the associations with incident CVD using models further adjusted for additional covariates (physical activity, smoking, drinking, BMI, hypertension, diabetes, dyslipidemia, SBP, and DBP) and evaluated the associations using separate models for each clinical risk factor at a time. The associations of biomarkers with incident CVD are generally preserved in the models (Additional file 1: Table S4).

### Population-attributable risk

**Fig. 4:**
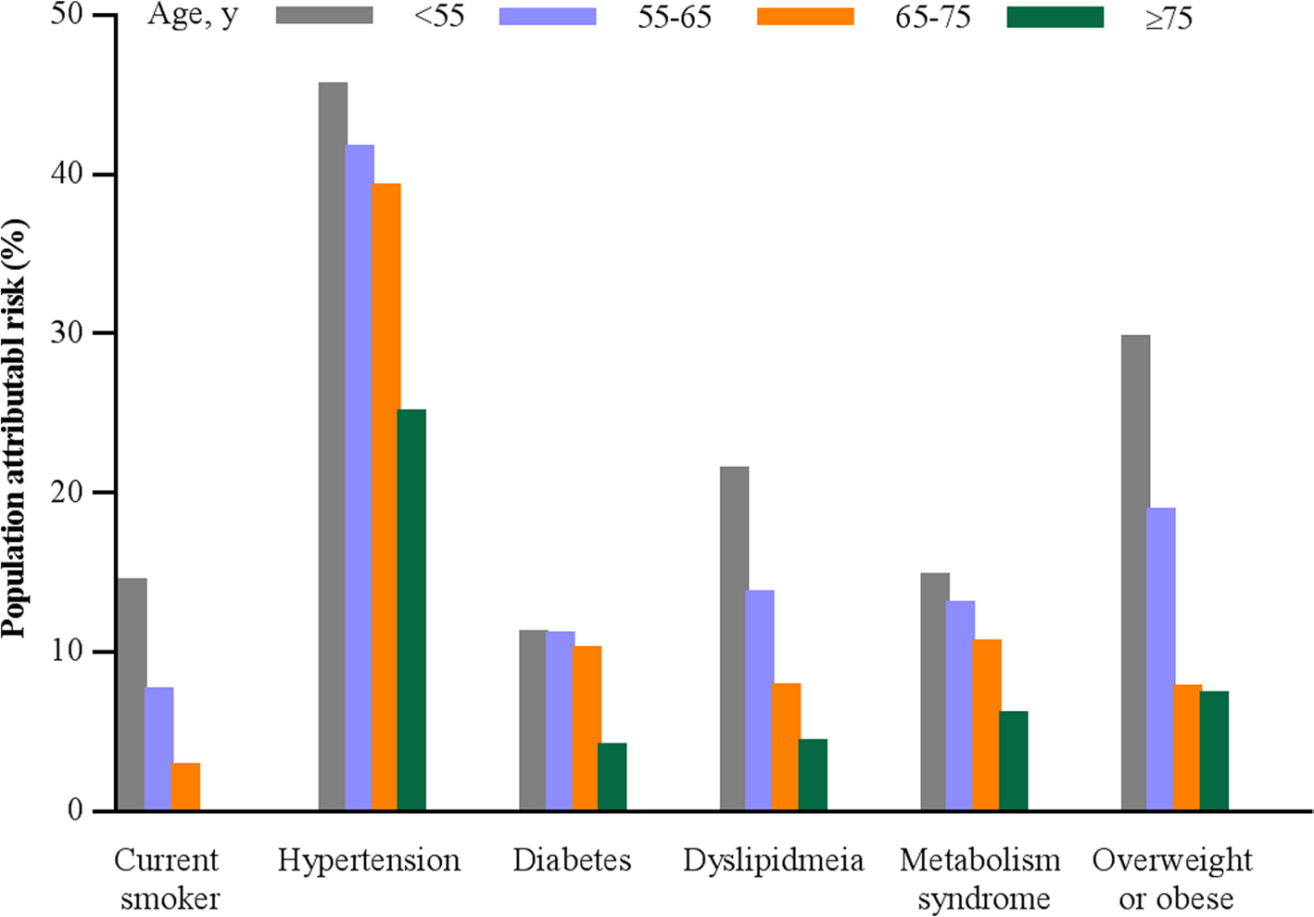
Population attribution risk for risk factors and incident cardiovascular disease across age categories

The population-attributable risk for the clinical risk factors attenuated with age (Fig. 4 and Additional file 1: Table S5). Of the categorical clinical risk factors analyzed, hypertension had the highest population-attributable risk in all age groups, which ranged from 45.4% (95% CI, 41.7–48.9%) for incident CVD onset younger than 55 years to 24.9% (95% CI, 19.0–30.7%) for onset in those 75 years or older, while smoking had the lowest population-attributable risk in all age groups.

## Discussion

In this large prospective study, we identified risk profiles associated with the risk of CVD occurring at younger ages. Of the clinical factors, diabetes was associated with the highest relative risk for incident CVD in participants younger than 55 years, in addition to hypertension, metabolism syndrome, overweight or obese, dyslipidemia, and smoking, which were also strong risk factors for premature CVD. Among the biomarkers examined in participants with CVD younger than 55 years, the TyG index reflecting insulin resistance had the highest magnitude of relative risk, which was greater than the association of FBG, lipids, or inflammatory biomarkers. Most clinical risk factors and biomarkers of cardiovascular risk showed age-attenuated associations with incident CVD. These findings underscore the importance of diabetes and insulin resistance as major determinants of premature CVD, as well as other modified major risk factors that can be addressed with lifestyle or preventive interventions.

Early life exposure of cardiovascular risk factors has been emerging as potentially important risk factor for premature CVD. Diabetes mellitus has been reported to be associated with excess cardiovascular morbidity and mortality in all age groups, while the most pronounced effect was observed in young people [19, 33]. A 23-year follow-up of the Da Qing Diabetes Study showed that young-onset diabetes was a risk factor for premature death and cardiovascular disease [33]. In line with prior findings, our study found that a history of diabetes was associated with a fourfold increase in the risk of premature CVD. Accumulating evidence supports that diabetes in younger people has a more rapid deterioration of β-cell function than is seen in later-onset diabetes [34]. This loss of β-cell function might result in higher SBP and LDL-C concentrations due to increased oxidative stress and activation of renin-angiotensin system, which would accelerate atherosclerosis and increase the risk of premature CVD [35]. Observations of blood pressure in young adults confirmed the effect of hypertension on CVD events [36]. Our data showed that a history of hypertension was associated with a 3.8-fold higher risk of premature CVD. Existing evidence suggested that early-onset hypertension has been found to be more affected by hereditary susceptibility [37]. Similarly, obese and overweight were associated with incident CVD and could be targeted to reduce the risk of CVD, in particular younger adults. Behavioral modification could also target smoking, as it was associated with a higher risk of incident CVD, especially in young adults. Smoking remains a major public health problem across all ages but in particular for younger individuals, and smoking cessation initiatives should remain part of efforts to reduce cardiovascular risk across all ages [38]. It should be noted that, in our study, current drinking tended to have protective effects on the risk of premature CVD, which was supported by several previous studies [39–41], demonstrating that alcohol consumption, especially moderate drinking was associated with decreased risk of CVD. Possible mechanisms may be that alcohol has been shown to raise HDL-C and apolipoprotein A-I, which are inversely associated with the risk of CVD. Additionally, alcohol consumption may also influence atherosclerotic plaque composition and provide stabilizing benefits.

Many previous studies have evaluated the associations between cholesterol levels and CVD risk in young adults. Studies on dyslipidemia and premature CVD measured biomarkers at the time of the acute CVD event and reported on the prevalence of dyslipidemia or risk of CVD based on unadjusted models, reaching disparate conclusions. These studies showed mixed results with a higher, lower, or similar prevalence of dyslipidemia in younger vs older adults [42–44]. Three large cohorts of younger men from the Chicago Heart Association Detection Project in Industry, Chicago People Gas Company, and Multiple Risk Factor Intervention Trial studies demonstrate a continuous, graded relationship of serum cholesterol level to long-term risk of CVD, substantial absolute risk, and absolute excess risk of CVD death for younger men with elevated serum cholesterol levels [45]. Data from the Coronary Artery Risk Development In Young Adults study indicated that non-optimal HDL-C at commonly observed levels during young adulthood was independently associated with coronary atherosclerosis two decades later [46]. Our present results are consistent with these previous observations and provide additional evidence for the atherogenicity of serum lipids (TC, TG, HDL-C, non-HDL-C, and remnant cholesterol) at different ages, and the effects were more pronounced in young adults.

In the present study, insulin resistance measured by the TyG index had the strongest association with premature CVD out of approximately 10 biomarkers examined. Insulin resistance is a chronic disorder that leads to deleterious changes in the blood vessel wall and premature CVD; the TyG index developed from TG and FBG was a simple and reliable surrogate for insulin resistance and was highly correlated with the hyperinsulinemic-euglycemic clamp and homeostasis model assessment of insulin resistance [20]. In our study, the TyG index had a greater association with CVD occurring in participants at younger ages and up to 75 years than all the other biomarkers. The TyG index potentially links insulin resistance and its concomitant thermogenic dyslipidemia with future risk of both diabetes and premature CVD. Chronic kidney disease is an emerging public health problem and can be regarded as a premature CVD entity [47]; our study showed that decreased eGFR and increased SUA were significantly associated with the risk of CVD, especially among young adults. This finding was supported by a previous study that hyperuricemia at an early age was associated with a higher risk of CVD than later-onset hyperuricemia [48].

The positive associations of inflammatory biomarkers with CVD, which was more pronounced for premature CVD, were supported by the growing evidence on the role of inflammation in initial and recurrent cardiovascular events. The Health, Aging and Body Composition study showed hs-CRP is less predictive of CVD in older compared with younger adults [49]. Lower platelet counts were reported to be associated with increased risk of thrombotic events, our study found the association between platelet counts and CVD differed by age of CVD onset, and increased platelet counts may be a protective role in CVD among adults with 55 to 65 years of CVD onset. In addition, neutrophil counts, as a ubiquitous biomarker of acute infection and inflammation, were strongly associated with the incidence of CVD. The findings were extended via our study by showing the positive association between neutrophil counts was more evident in younger adults. Several cardiovascular disease risk factors were associated with high inflammation levels, including smoking, blood pressure, diabetes, BMI, and abdominal adiposity [50], suggesting that inflammation that accompanies excess adiposity states, such as diabetes and insulin resistance, could be even more relevant for CVD occurring at a younger age.

In this study, the incidence rates of CVD increased with age, consistent with age being a substantial risk factor. Analysis of the relative rates of risk factors for incident CVD should not, however, imply that risk factors are more important at younger vs older ages. Therefore, the importance of primary CVD prevention among older adults is not diminished by the observed attenuation of relative rates of risk factors with incident CVD in older individuals. Rather, these results suggest a stronger relative association of risk factors with younger vs older ages and emphasize the need for improved primary prevention among younger adults. The age-related attenuation of relative risk has implications for cardiovascular risk modeling depending on the age group in which CVD occurs. Young adults are unaware of their heightened cardiometabolic and mortality risk, which would still translate into a great disease burden. Improving modifiable clinical risk factors could substantially reduce CVD risk.

Strengths of this study included the large number of participants, long follow-up period, and the collection of information on various lifestyle factors and biomarkers. The study also has several limitations. First, lifestyle and medical history were self-reported, which may be subject to recall bias. Second, there are challenges to compare the risks associated with clinical categorical variables vs continuous biomarkers. Similar issues affect the population-attributable risks, which depend on the magnitude of risk association with the prevalence of the risk factors in the population. Third, our study was conducted among Chinese; thus, the results may not be generalized to other populations.

## Conclusions

In conclusion, our study has identified risk factors and biomarkers associated with the risk of CVD occurring in adults at younger vs older ages. The most substantial risk of premature CVD was associated with diabetes and insulin resistance biomarkers, as well as hypertension, metabolic syndrome, overweight or obese, dyslipidemia, and smoking. Most lipid profiles and metabolic and inflammatory biomarkers were also associated with premature CVD risk, albeit their relative magnitude was less than the TyG index. Although the relative risk of CVD was attenuated with age, cardiometabolic risk factors prevention and management remained important at all ages. These findings highlight the importance of early identification, screening, stratification, and treatment to decrease the burden of premature CVD and mortality.

## Supplementary Information

**Additional file 1: Table S1.** Baseline characteristics of participants with and without incident CVD. **Table S2.** Baseline characteristics of participants by different age. **Table S3.** Incidence rate of CVD in different age group. **Table S4.** Multivariable-adjusted associations of risk factors with incident CVD by age at onset. **Table S5.** PAR and 95% CI for cardiovascular disease by risk factors.

## Data Availability

Data are available to researchers on request for purposes of reproducing the results or replicating the procedure by directly contacting the corresponding author.

## Abbreviations

BMI: Body mass index
CI: Confidence interval
CVD: Cardiovascular disease
DBP: Diastolic blood pressure
eGFR: Estimated glomerular filtration rate
FBG: Fasting blood glucose
HDL-C: High-density lipoprotein cholesterol
hs-CRP: High-sensitivity C-reactive protein
LDL-C: Low-density lipoprotein cholesterol
SBP: Systolic blood pressure
SD: Standard deviation
sHR: Sub-distributional hazard ratio
TC: Total cholesterol
TG: Triglyceride
TyG index: Triglyceride-glucose index
