# Supplementary material for: Association of lipid, inflammatory, and metabolic biomarkers with age at onset for incident cardiovascular disease
Source: BMC Med. 2022 Nov 10;20:383. doi: 10.1186/s12916-022-02592-x (PMC9647925; doi:10.1186/s12916-022-02592-x)
Supplement: Supplementary file 1 — Additional file 1: Table S1. Baseline characteristics of participants with and without incident CVD. Table S2. Baseline characteristics of participants by different age. Table S3. Incidence rate of CVD in different age group. Table S4. Multivariable-adjusted associations of risk factors with incident CVD by age at onset. Table S5. PAR and 95% CI for cardiovascular disease by risk factors. [file 12916_2022_2592_MOESM1_ESM.docx]

**SUPPLEMENTAL MATERIALS**

Table S1. Baseline characteristics of participants with and without incident CVD

| Characteristics | Non-cases | Incident CVD | *P* value |
| --- | --- | --- | --- |
| Clinical risk factors |  |  |  |
| Age, y | 50.96±12.59 | 58.13±10.37 | <0.0001 |
| Men, n (%） | 71048 (78.77) | 6770 (89.23) | <0.0001 |
| High school or above, n (%) | 6389 (7.37) | 213 (2.99) | <0.0001 |
| Income >800 yuan/month, n (%) | 12427 (14.34) | 884 (12.42) | <0.0001 |
| Current smoker, n (%) | 29577 (33.85) | 2751 (38.18) | <0.0001 |
| Current drinker, n (%) | 32760 (37.48) | 2630 (36.51) | 0.1039 |
| Physical inactivity, n (%) | 7631 (8.82) | 526 (7.41) | 0.0001 |
| Hypertension, n (%) | 36969 (40.99) | 4956 (65.32) | <0.0001 |
| Diabetes, n (%) | 7438 (8.25) | 1304 (17.19) | <0.0001 |
| Dyslipidmeia, n (%) | 31145 (34.53) | 3233 (42.61) | <0.0001 |
| Metabolism syndrome, n (%) | 11967 (13.27) | 1790 (23.59) | <0.0001 |
| Body mass index, kg/m^2^ | 24.96±3.49 | 25.70±3.47 | <0.0001 |
| Overweight or obese, n (%) | 42098 (46.67) | 4209 (55.48) | <0.0001 |
| Systolic blood pressure, mm Hg | 129.64±20.41 | 142.37±22.82 | <0.0001 |
| Diastolic blood pressure, mm Hg | 82.96±11.58 | 88.23±12.83 | <0.0001 |
| Lipids profile |  |  |  |
| Total cholesterol, mmol/L | 4.93±1.14 | 5.11±1.19 | <0.0001 |
| Triglycerides, mmol/L | 1.66±1.36 | 1.88±1.49 | <0.0001 |
| LDL cholesterol, mmol/L | 2.34±0.90 | 2.39±1.03 | <0.0001 |
| HDL cholesterol, mmol/L | 1.55±0.40 | 1.56±0.43 | 0.0009 |
| Total/HDL cholesterol | 3.39±3.26 | 3.48±1.36 | 0.0146 |
| Triglyceride/HDL cholesterol | 1.17±2.26 | 1.31±1.31 | <0.0001 |
| Non-HDL-C, mmol/L | 3.38±1.11 | 3.55±1.18 | <0.0001 |
| Remnant cholesterol, mmol/L | 1.04±1.15 | 1.15±1.28 | <0.0001 |
| Metabolic and Inflammatory |  |  |  |
| Fasting blood glucose, mmol/L | 5.43±1.61 | 5.91±2.19 | <0.0001 |
| Triglyceride-glucose index | 8.64±0.69 | 8.83±0.72 | <0.0001 |
| eGFR, ml/min/1.73/m^2^ | 82.56±25.47 | 78.77±28.92 | <0.0001 |
| Creatinine, μmol/L | 91.56±29.85 | 94.48±37.71 | <0.0001 |
| Serum uric acid, μmol/L | 287.85±83.18 | 303.15±88.81 | <0.0001 |
| Hs-CRP, mg/L | 2.33±6.48 | 3.04±6.35 | <0.0001 |
| White blood cell count, *10^9/L | 6.83±9.86 | 6.99±7.46 | 0.1876 |
| Neutrophil count, *10^9/L | 3.97±2.91 | 4.14±1.84 | <0.0001 |
| P[latelet](http://www.baidu.com/link?url=zM45hDoKDddfCCOQu7hZG5idQKqfIJinJ2kmxS_sjWE7yuml8t0MAlgoPXgk-AIopgc5PpCWpVwyikNHl5F_yYW9PUwHyobO93ZinyR1UQf-8zzFrJ12urGMtnFSl5Cf), *10^9/L | 211.22±746.16 | 202.92±73.04 | 0.3413 |
| Red blood cell count, *10^9/L | 5.05±26.46 | 5.00±8.36 | 0.9004 |

Abbreviations: eGFR, estimated glomerular filtration rate; HDL, high density lipoprotein; hs-CRP, high sensitivity C-reactive protein; LDL, low density lipoprotein.

Table S2. Baseline characteristics of participants by different age

| Characteristics | At age<55 y | At 55 to <65 y | At 65 to <75 y | At age ≥75 y | *P* value |
| --- | --- | --- | --- | --- | --- |
| Clinical risk factors |  |  |  |  |  |
| Age, y | 34.84±6.55 | 48.23±3.86 | 57.13±3.93 | 70.49±6.12 | <0.0001 |
| Men, n (%） | 16324 (75.44) | 24426 (77.21) | 23104 (81.19) | 13964 (86.98) | <0.0001 |
| High school or above, n (%) | 3997 (18.61) | 1286 (4.16) | 767 (2.83) | 552 (3.86) | <0.0001 |
| Income >800 yuan/month, n (%) | 3839 (17.88) | 3464 (11.20) | 3731 (13.78) | 2277 (15.97) | <0.0001 |
| Current smoker, n (%) | 8092 (37.60) | 11597 (37.30) | 9031 (33.17) | 3608 (24.47) | <0.0001 |
| Current drinker, n (%) | 9865 (45.83) | 11970 (38.50) | 9283 (34.08) | 4272 (28.94) | <0.0001 |
| Physical inactivity, n (%) | 2436 (11.35) | 3278 (10.60) | 1808 (6.693) | 635 (4.466) | <0.0001 |
| Hypertension, n (%) | 5320 (24.59) | 12922 (40.85) | 14138 (49.68) | 9545 (59.45) | <0.0001 |
| Diabetes, n (%) | 822 (3.80) | 2823 (8.924) | 3128 (10.99) | 1969 (12.26) | <0.0001 |
| Dyslipidemia, n (%) | 6609 (30.54) | 11324 (35.80) | 10983 (38.60) | 5462 (34.02) | <0.0001 |
| Metabolism syndrome, n (%) | 1499 (6.93) | 4258 (13.46) | 5042 (17.72) | 2958 (18.42) | <0.0001 |
| Body mass index, kg/m^2^ | 24.75±3.78 | 25.13±3.35 | 25.26±3.34 | 24.72±3.58 | <0.0001 |
| Overweight or obese, n (%) | 9538 (44.08) | 15423 (48.75) | 14225 (49.99) | 7121 (44.35) | <0.0001 |
| Systolic blood pressure, mm Hg | 121.13±17.47 | 129.07±19.80 | 134.17±20.93 | 140.33±21.35 | <0.0001 |
| Diastolic blood pressure, mm Hg | 80.07±11.52 | 84.18±11.99 | 85.03±11.60 | 83.33±11.01 | <0.0001 |
| Lipids profile |  |  |  |  |  |
| Total cholesterol, mmol/L | 4.75±1.11 | 5.00±1.12 | 5.04±1.17 | 4.93±1.17 | <0.0001 |
| Triglycerides, mmol/L | 1.63±1.41 | 1.74±1.47 | 1.71±1.36 | 1.54±1.11 | <0.0001 |
| LDL cholesterol, mmol/L | 2.37±0.77 | 2.36±0.86 | 2.35±1.00 | 2.27±1.02 | <0.0001 |
| HDL cholesterol, mmol/L | 1.49±0.35 | 1.54±0.38 | 1.57±0.42 | 1.61±0.46 | <0.0001 |
| Total/HDL cholesterol | 3.36±2.48 | 3.41±1.53 | 3.45±3.68 | 3.31±4.89 | <0.0001 |
| Triglyceride/HDL cholesterol | 1.20±3.99 | 1.21±1.18 | 1.19±1.32 | 1.06±1.44 | <0.0001 |
| Non-HDL-C, mmol/L | 3.26±1.07 | 3.46±1.09 | 3.47±1.15 | 3.32±1.15 | <0.0001 |
| Remnant cholesterol, mmol/L | 0.89±1.06 | 1.10±1.13 | 1.12±1.22 | 1.05±1.23 | <0.0001 |
| Metabolic and Inflammatory |  |  |  |  |  |
| Fasting blood glucose, mmol/L | 5.21±1.25 | 5.52±1.68 | 5.57±1.80 | 5.53±1.85 | <0.0001 |
| Triglyceride-glucose index | 8.56±0.71 | 8.69±0.71 | 8.70±0.68 | 8.61±0.65 | <0.0001 |
| eGFR, ml/min/1.73/m^2^ | 91.36±26.67 | 83.81±24.67 | 79.64±23.08 | 71.58±26.46 | <0.0001 |
| Creatinine, μmol/L | 92.07±33.05 | 91.57±31.86 | 90.67±26.74 | 93.82±30.54 | <0.0001 |
| Serum uric acid, μmol/L | 285.49±82.46 | 281.49±80.74 | 290.18±83.31 | 306.82±89.16 | <0.0001 |
| Hs-CRP, mg/L | 1.79±4.82 | 2.07±6.68 | 2.60±5.99 | 3.48±8.49 | <0.0001 |
| White blood cell count, *10^9/L | 6.86±3.89 | 6.94±10.89 | 6.81±10.4 | 6.7±11.15 | 0.0840 |
| Neutrophil count, *10^9/L | 4.00±2.28 | 4.03±2.78 | 3.94±2.42 | 3.95±4.04 | 0.0022 |
| P[latelet](http://www.baidu.com/link?url=zM45hDoKDddfCCOQu7hZG5idQKqfIJinJ2kmxS_sjWE7yuml8t0MAlgoPXgk-AIopgc5PpCWpVwyikNHl5F_yYW9PUwHyobO93ZinyR1UQf-8zzFrJ12urGMtnFSl5Cf), *10^9/L | 226.32±1340.95 | 213.22±140.58 | 202.98±155.81 | 197.88±810.81 | 0.0004 |
| Red blood cell count, *10^9/L | 5.17±31.11 | 4.88±4.22 | 4.95±7.25 | 5.43±54.42 | 0.3189 |

Table S3. Incidence rate of CVD in different age group

|  | Incident CVD, incident rate (95%CI) | | | |
| --- | --- | --- | --- | --- |
|  | At age <55 y | At age 55 to 65 y | At age 65 to 75 y | At age ≥75 y |
| Total population | 4.69(4.44-4.96) | 6.59(6.33-6.87) | 7.01(6.75-7.28) | 7.50(7.11-7.90) |

Abbreviations: CVD, cardiovascular disease; CI, confidence interval.

Table S4. Multivariable-adjusted associations of risk factors with incident CVD by age at onset

|  | Incident CVD, adjusted sHR (95%CI) | | | | *P* for interaction |
| --- | --- | --- | --- | --- | --- |
|  | At age <55 y | At age 55 to 65 y | At age 65 to 75 y | At age ≥75 y |  |
| Clinical risk factors |  |  |  |  |  |
| Current smoker | 1.36(1.19-1.56) | 1.15(1.05-1.27) | 1.03(0.93-1.14) | 1.01(0.80-1.04) | <0.0001 |
| Current drinker | 0.72(0.63-0.83) | 0.71(0.64-0.78) | 0.83(0.75-0.92) | 0.89(0.78-1.01) | 0.6488 |
| Physical inactivity | 1.18(1.03-1.35) | 1.07(0.94-1.28) | 1.08(0.94-1.28) | 1.06(0.98-1.19) | 0.1390 |
| Hypertension | 3.37(2.98-3.82) | 2.43(2.24-2.63) | 2.16(1.98-2.36) | 1.57(1.40-1.75) | <0.0001 |
| Diabetes | 3.71(3.15-4.37) | 2.22(2.01-2.46) | 2.02(1.81-2.24) | 1.33(1.15-1.54) | <0.0001 |
| Dyslipidemia | 1.48(1.32-1.66) | 1.27(1.17-1.37) | 1.18(1.09-1.29) | 1.10(0.99-1.23) | <0.0001 |
| Metabolism syndrome | 2.33(1.97-2.76) | 1.79(1.63-1.97) | 1.46(1.32-1.62) | 1.30(1.13-1.49) | <0.0001 |
| BMI, per SD increment | 1.22(1.16-1.27) | 1.18(1.14-1.22) | 1.06(1.01-1.11) | 1.10(1.04-1.15) | <0.0001 |
| Overweight or obese | 1.52(1.35-1.72) | 1.28(1.19-1.39) | 1.15(1.04-1.28) | 1.12(1.03-1.22) | <0.0001 |
| Systolic BP, per SD increment | 2.08(1.96-2.21) | 1.67(1.62-1.73) | 1.55(1.49-1.60) | 1.23(1.18-1.29) | <0.0001 |
| Diastolic BP, per SD increment | 1.79(1.71-1.88) | 1.43(1.38-1.48) | 1.25(1.20-1.30) | 1.08(1.02-1.14) | <0.0001 |
| Lipids, per SD increment |  |  |  |  |  |
| Total cholesterol | 1.23(1.17-1.28) | 1.06(1.02-1.09) | 1.04(1.00-1.09) | 1.07(1.02-1.13) | <0.0001 |
| Triglycerides | 1.03(0.98-1.08) | 1.01(0.98-1.05) | 1.01(0.97-1.05) | 1.06(0.99-1.13) | 0.0044 |
| LDL cholesterol | 1.03(0.96-1.11) | 1.01(0.97-1.06) | 1.03(0.99-1.07) | 1.02(0.98-1.07) | 0.5457 |
| HDL cholesterol | 1.14(1.08-1.20) | 1.06(1.02-1.10) | 1.05(1.02-1.10) | 0.99(0.94-1.04) | 0.0090 |
| Total/HDL cholesterol | 1.00(0.98-1.02) | 1.01(0.96-1.05) | 0.99(0.95-1.02) | 1.01(0.99-1.02) | 0.7425 |
| Triglycerides/HDL cholesterol | 0.99(0.96-1.01) | 1.00(0.94-1.07) | 1.00(0.93-1.08) | 1.02(0.99-1.06) | 0.8310 |
| Non-HDL cholesterol | 1.22(1.16-1.28) | 1.04(1.00-1.08) | 1.02(0.98-1.06) | 1.07(1.02-1.13) | <0.0001 |
| Remnant cholesterol | 1.21(1.15-1.27) | 1.03(0.99-1.07) | 1.00(0.96-1.04) | 1.04(0.99-1.10) | <0.0001 |
| Metabolic, per SD increment |  |  |  |  |  |
| Fasting blood glucose | 1.14(1.08-1.20) | 1.08(1.04-1.12) | 1.00(0.95-1.04) | 1.05(0.99-1.11) | <0.0001 |
| Triglyceride-glucose index | 1.20(1.13-1.29) | 1.12(1.08-1.18) | 1.05(1.00-1.10) | 1.11(1.03-1.18) | <0.0001 |
| eGFR | 0.81(0.76-0.86) | 0.82(0.77-0.87) | 0.76(0.69-0.83) | 0.97(0.86-1.09) | <0.0001 |
| Creatinine | 0.97(0.91-1.04) | 1.02(0.99-1.05) | 1.06(1.02-1.10) | 1.04(1.00-1.08) | 0.0827 |
| Serum uric acid | 1.12(1.06-1.19) | 1.11(1.06-1.15) | 1.20(1.15-1.25) | 1.08(1.03-1.13) | 0.0068 |
| Inflammatory, per SD increment |  |  |  |  |  |
| Hs-CRP | 1.12(1.08-1.16) | 1.03(1.01-1.05) | 1.05(1.02-1.07) | 1.03(1.00-1.05) | 0.0009 |
| White blood cell count | 1.05(1.00-1.11) | 1.00(0.99-1.01) | 1.02(0.99-1.05) | 0.97(0.93-1.01) | 0.3529 |
| Neutrophil count | 1.06(1.03-1.09) | 1.02(1.01-1.04) | 1.05(1.02-1.08) | 0.99(0.96-1.03) | 0.0663 |
| P[latelet](http://www.baidu.com/link?url=zM45hDoKDddfCCOQu7hZG5idQKqfIJinJ2kmxS_sjWE7yuml8t0MAlgoPXgk-AIopgc5PpCWpVwyikNHl5F_yYW9PUwHyobO93ZinyR1UQf-8zzFrJ12urGMtnFSl5Cf) | 1.00(0.99-1.00) | 0.52(0.35-0.79) | 0.80(0.45-1.40) | 0.98(0.88-1.09) | 0.0223 |
| Red blood cell count | 0.00(0.00-0.00) | 1.01(0.98-1.03) | 1.01(0.98-1.05) | 0.97(0.94-1.00) | 0.8380 |

sHR (95% CI) were obtained from stratified competing risk models with non-CVD death as a competing risk, adjusted for gender, educational level and family income, and interactions between the risk factors of interest and age groups.

Abbreviation: sHR, sub-distributional hazard ratio.

Table S5. PAR and 95% CI for cardiovascular disease by risk factors

| Clinical risk factors | PAR (95% CI) | | | |
| --- | --- | --- | --- | --- |
|  | At age <55 y | At age 55 to 65 y | At age 65 to 75 y | At age ≥75 y |
| Current smoker | 14.3 (9.9-18.7) | 7.4 (4.5-10.4) | 2.7 (-0.1-5.6) | - |
| Hypertension | 45.4 (41.7-48.9) | 41.5 (38.5-44.5) | 39.1 (35.4-42.8) | 24.9 (19.0-30.7) |
| Diabetes | 11.0 (9.0-13.0) | 10.9 (9.3-12.5) | 10.0 (8.2-11.8) | 3.9 (1.8-6.1) |
| Dyslipidemia | 21.3 (17.4-25.2) | 13.5 (10.6-16.4) | 7.7 (4.4-11.0) | 4.2 (0.3-8.0) |
| Metabolism syndrome | 14.6 (12.2-16.9) | 12.9 (11.0-14.7) | 10.4 (8.2-12.5) | 5.9 (3.1-8.6) |
| Overweight or obese | 29.5 (24.6-34.3) | 18.7 (15.1-22.3) | 7.6 (3.5-11.7) | 7.2 (2.4-11.9) |

Abbreviations: CI, confidence interval; PAR, population attributable risk
